# Supplementary material for: Comparison and correlation of cervical proprioception and muscle endurance in general joint hypermobility participants with and without non-specific neck pain—a cross-sectional study
Source: PeerJ. 2022 Mar 11;10:e13097. doi: 10.7717/peerj.13097 (PMC8919848; doi:10.7717/peerj.13097)
Supplement: Supplemental Information 1 [file peerj-10-13097-s001.docx]

| Group | 1=nonspecific neck pain, 2= Asymptomatic subjects |
| --- | --- |
| AGE | In years |
| Gender | 1=male, 2= female |
| HEIGHT | In meters |
| WEIGHT | kg |
| BMI | Weight/height^2^ |
| Beighton score | Hypermobility score |
| VAS | Neck pain intensity in Visual analogue scale (1-10cm) |
| NDI | Neck disability index (0-50 score) |
| THP_Flex | Target head position in flexion (degrees) |
| THP_Ext | Target head position in extension (degrees) |
| THP_RL | Target head position in rotation left (degrees) |
| THP_RR | Target head position in rotation right (degrees) |
| NFE | Neck flexor endurance in sec |
| NEE | Neck extensor endurance in sec |
